# Referral reasons for evaluating childhood glaucoma in a tertiary service

**DOI:** 10.5935/0004-2749.20220067

**Published:** 2025-08-22

**Authors:** André Leite, Christiane Rolim-de-Moura

**Affiliations:** 1 Universidade Federal de São Paulo, São Paulo, SP, Brazil

**Keywords:** Glaucoma/congenital, Glaucoma/physiopathology, Corneal opacity, Child, Visual acuity, Referral and consultation, Eye health services, Glaucoma/congênito, Glaucoma/fisiopatologia, Opa cidade da córnea, Criança, Acuidade visual, Encaminhamento e consulta, Serviços de saúde ocular

## Abstract

**Purpose:**

To report the distribution of referral reasons for children from a pediatric glaucoma outpatient clinic in a tertiary eye care service.

**Methods:**

The medical records of patients aged <18 years who were referred to a pediatric glaucoma center in the city of São Paulo, Brazil, between 2012 and 2018 were retrospectively reviewed. The data collected included the referral reasons, age, hospital of origin, and who detected the ocular alteration. For defining the diagnosis, the Childhood Glaucoma Research Network classification was used.

**Results:**

Five hundred sixty-three eyes of 328 patients were included in the study. Glaucoma diagnosis was confirmed in 162 children (49%). In 83 patients (25%), the glaucoma diagnosis was ruled out, and 83 (25%) continued outpatient follow-up for suspected glaucoma. The main referral reasons were a cup-to-disc ratio >0.5 or an asymmetry ≥0.2 (24%), intraocular pressure >21 mmHg (21%), and corneal opacity (15%). In the neonatal period, the referral reasons were corneal opacity, buphthalmos, tearing, and photophobia. After the neonatal period, besides these external changes, other signs were also reasons for referral, such as cup-to-disc ratio >0.5 or asymmetry ≥0.2, intraocular pressure >21 mmHg, and myopic shift. The referrals were made by health professionals in 69% and parental concern in 30% of the cases. In 97% of the primary congenital glaucoma cases, the parents were the first to identify the change and to seek for health care.

**Conclusions:**

The referral reasons of the children to a tertiary glaucoma clinic were differed between the age groups and diagnoses. We suggest that awareness with these findings is important to avoid and postpone diagnosis, identify their impacts on prognosis, and avoid spending important resources for the management of diseases with inaccurate referrals.

## Abstract (pt)

**Objetivos:**

Relatar a distribuição dos motivos de encaminhamento de crianças para ambulatório de glaucoma infantil em um serviço oftalmológico terciário.

**Métodos:**

Dados médicos de pacientes menores que 18 anos encaminhados para ambulatório de glaucoma infantil na cidade de São Paulo, Brasil, de 2012 a 2018 foram retrospectivamente analisados. Os dados incluíram os motivos de encaminhamento, a idade, a origem e quem detectou a alteração ocular. Para definição diagnóstica, a classificação do *Childhood Glaucoma Research Network* foi usada.

**Resultados:**

563 olhos de 328 pacientes foram incluídos no estudo. O diagnóstico de glaucoma foi confirmado em 162 crianças (49%). 83 (25%) pacientes tiveram diagnóstico de glaucoma descartado, e 83 (25%) continuaram em acompanhamento como glaucoma suspeito. Os principais motivos de encaminhamento foram relação escavação-disco >0,5 ou assimetria ≥0,2 (24%), pressão intraocular >21 mmHg (21%) e opacidade corneana (15%). No período neonatal, os motivos de encaminhamento foram opacidade corneana, buftalmo, lacrimejamento e fotofobia. Após o período neonatal, além desses sinais externos, outros sinais foram também motivos de encaminhamento, como escavação-disco >0,5 ou assimetria ≥0,2, pressão intraocular >21 mmHg e miopização. Os encaminhamentos ocorreram por profissionais de saúde em 69% e preocupação dos pais em 30%. Os pais foram os primeiros a identificar as alterações e procurar cuidado médico em 97% dos casos de glaucoma congênito primário.

**Conclusões:**

Os motivos de encaminhamento de crianças para um serviço de glaucoma de glaucoma terciário foram determinados e diferem em diferentes faixas etárias e grupos. Os autores reforçam a necessidade de alertar diferentes grupos para os sinais mais comuns, a fim de evitar, não só o adiamento do diagnóstico, o que impacta no prognóstico, mas também despender recursos importantes direcionados ao enfrentamento dessas doenças, com encaminhamentos imprecisos.

## INTRODUCTION

Childhood glaucoma encompasses a heterogeneous group of diseases that may lead to irreversible vision loss, which compromises school performance and quality of life^(1,2)^. The prevalence and distribution of glaucoma in the pediatric population vary among studies. The mean prevalence rates of low visual acuity due to glaucoma in the pediatric population range from 3.9% in the European region to 10.9% in the African region^(3)^. At 1 year of age, the annual incidence of primary congenital glaucoma ranges from 5.41 per 100,000 live births (1/18,500) in Britain and 3.31 per 100,000 live births (1/30,200) in the Republic of Ireland to 1 per 3,030 live births in Saudi Arabia^(4,5)^.

In a retrospective study conducted in Brazil with 3,210 children with low visual acuity, 10.8% of the cases were due to glaucoma^(6)^. In another Brazilian study, 69% of children with congenital glaucoma according to the best-corrected visual acuity in the better eye presented with a visual acuity worse than 20/63 (28% moderately low visual acuity; 15% severely low visual acuity; 11% profoundly low visual acuity, and 15% near blindness). Of the school-aged children, 69.3% had optical aids prescribed for distance vision and 12.2% had optical aids prescribed for near vision^(7)^.

Owing to the rarity of childhood glaucoma, most suspected cases are referred to a tertiary service for diagnostic investigation, management, and follow-up^(4)^. In some cases, parents or caregivers are the first for detect the eye changes and to seek for health care. In other cases, an ocular change is perceived by routine screening by a health-care professional^(4)^. Knowledge of the main signs and referral reasons of children with suspected glaucoma may aid in identifying and treating the early symptoms of glaucoma and, consequently, minimize sequelae.

The aim of this study was to evaluate the distribution of referral reasons for children from a pediatric glaucoma outpatient clinic in a tertiary health-care service.

## METHODS

The medical records of patients aged <18 years who were referred to the pediatric glaucoma center of the Department of Ophthalmology and Visual Sciences of Federal University of São Paulo in the city of São Paulo, Brazil, between 2012 and 2018 were retrospectively reviewed. Only patients aged <18 years with referral information in their medical records or telephone contact were included in the study. We recorded the reasons, age, hospital origin of the referral, and person who detected the ocular alteration, and clinical and family health histories of each patient. In cases with >1 referral reason for the same patient, all the identified reasons were recorded. This study was approved by the research ethics committee of São Paulo Hospital (approval No. 2.967.795) and was compliant with the principles of the Declaration of Helsinki. Parental consent was obtained for all the patients.

**Figure 1:**
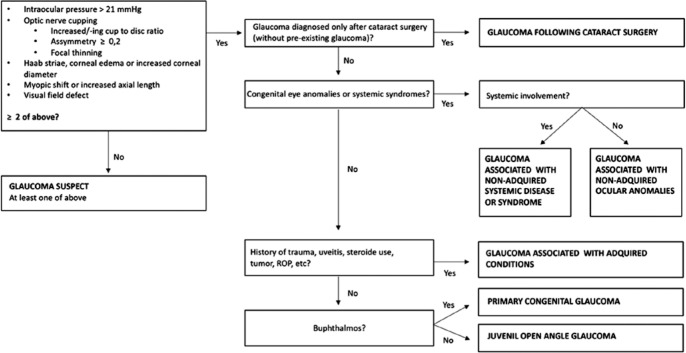
Diagnostic criteria for childhood glaucoma, based on Childhood Glaucoma Research Network (CGRN) classification^(8)^

The patients were diagnosed and classified as having suspected glaucoma or glaucoma using the Childhood Glaucoma Research Network (CGRN) classification (Figure 1)^(8)^. Patients who did not meet the criteria for suspected glaucoma or glaucoma were classified as having no glaucoma. Patients were further classified according to the onset of symptoms. Glaucoma was classified as neonatal (until 30 days of life), infantile (between 30 days and 2 years of life), or late manifestation (after 2 years of life). This categorization into neonatal, infantile, or late manifestation accounts for the time the signs appeared and may or may not coincide with the time of referral.

We classified the referral as child health surveillance/ screening when the child was referred by health caregivers (pediatricians, nurses, and general ophthalmologists) in a routine evaluation; clinical surveillance when a general ophthalmologist refers a child with a known ocular disease that is at risk of developing glaucoma; and parental concern when parents or caregivers were the first to detect the eye abnormalities and seek for health care (at first with a general ophthalmologist and then with a tertiary center with the childhood glaucoma consultants).

The data obtained were analyzed using the STATA 14.0 program (StataCorp LP, College Station, TX, USA). Categorical data are presented as counts and percentages, and continuous data are presented as means and standard deviations (range).

## RESULTS

Of the 575 patients referred during the study period, 328 (57%) met the inclusion criteria and 328 (563 eyes) were included in the study. One hundred fifty children (46%) were female, with a mean referral age of 55 ± 49 months (range, 0-215 months). Table 1 shows the distribution of each glaucoma group according to the number and percentage of children, mean age at the time of referral, sex, and laterality. In the 328 referred patients, follow-up for suspected glaucoma. In the patients with a we confirmed the glaucoma diagnosis in 162 children final diagnosis of glaucoma, the most common diagnosis (49%). In 83 patients (25%), the glaucoma diagnosis was was primary congenital glaucoma (20%), followed by ruled out, and 83 patients (25%) continued outpatient glaucoma following cataract surgery (12%).

**Table 1:** Distribution of each glaucoma group according to number of children, mean age of referral, gender and laterality

Final diagnosis	No. of children n (%)	Age at referral in months mean ± SD (range)	Sex, female n (%)	Bilateral n (%)	Unilateral - right eye n (%)	Unilateral - left eye n (%)	Total, eyes n (%)
**GS**	83 (25)	75 ± 43 (1-194)	34 (41)	66 (79)	6 (7.2)	11 (13)	149 (100)
**PCG**	66 (20)	9.2 ± 13 (0-72)	25 (38)	50 (76)	8 (12)	8 (12)	116 (100)
**GFCS**	38 (12)	63 ± 47 (1-168)	20 (53)	19 (50)	11(28)	8 (21)	57 (100)
**JOAG**	2 (0.6)	106 ± 45 (74-138)	1 (50)	2 (100)	0	0	4 (100)
**GAAC**	20 (6.1)	106 ± 70 (24-215)	10 (50)	9 (45)	6 (30)	5 (25)	29 (100)
**GNAOA**	27 (8.2)	31 ± 33 (0-127)	16 (59)	20 (74)	3 (11)	4 (15)	47 (100)
**GNAS**	9 (2.7)	45 ± 46 (0-96)	4 (44)	4 (44)	0	5 (56)	13 (100)
**No glaucoma**	83 (25)	63 ± 40 (0-187)	40 (48)	65 (78)	10 (12)	8 (10)	148 (100)
**Total**	**328 (100)**	**55 ± 49 (0-215)**	**150 (46)**	**235 (72)**	**44 (13)**	**49 (15)**	**563 (100)**

SD= Standard deviation; GS= glaucoma suspect; PCG= Primary congenital glaucoma; JOAG= Juvenile open angle glaucoma; GNAOA= Glaucoma associated with non-acquired ocular anomalies; GAAC= Glaucoma associated with acquired conditions; GFCS= Glaucoma following cataract surgery; GNAS= Glaucoma associated with non-acquired systemic disease or syndrome

Table 2 shows the referral reasons in each group of patients with glaucoma. From among the 328 referred patients, 397 referral reasons were identified as follows: 94 (24%), cup-to-disc ratio (CDR) >0.5 or asymmetry ≥0.2; 82 (21%), intraocular pressure (IOP) >21 mmHg; 60 (15%), corneal opacity; 57 (14%), buphthalmos; 34 (8.6%), conditions at risk of developing glaucoma; 30 (7.6%), tearing; 21 (5.3%), photophobia; 17 (4.3%), myopic shift; and 2 (0.5%), family history of glaucoma.

**Table 2:** Referral reasons according to glaucoma group

Referral reasons	No glaucoma n (%)	GS n (%)	PCG n (%)	JOAG n (%)	GNAOA n (%)	GAAC n (%)	GFCS n (%)	GNAS n (%)	Total number of children by each referral reason^a^	Children with confirmed glaucoma by each referral reason n (%)^b^
**Cup-to-disc ratio >0.5 or asymmetry** ≥**0.2**	39 (42)	47 (53)	0	2 (100)	1 (3.2)	1 (5.0)	3 (7.3)	1 (9.1)	94	8 (8.5)
**IOP >21 mmHg**	13 (13)	19 (21)	0	0	9 (29)	14 (70)	25 (61)	2 (18)	82	50 (61)
**Corneal opacity**	4 (4.3)	4 (4.5)	38 (34)	0	9 (29)	0	0	5 (45)	60	52 (87)
**Buphthalmos**	9 (9.8)	4 (4.5)	33 (30)	0	4 (13)	0	5 (12)	2 (18)	57	44 (77)
**Conditions at risk of developing glaucoma**	12 (13)	9 (10)	0	0	5 (16)	4 (20)	3 (7.3)	1 (9.1)	34	13 (38)
**Tearing**	3 (3.3)	1 (1.1)	25 (22)	0	1 (3.2)	0	0	0	30	26 (87)
**Photophobia**	5 (5.4)	0	15 (13)	0	1 (3.2)	0	0	0	21	16 (76)
**Myopic shift**	6 (6.5)	4 (4.5)	0	0	1 (3.2)	1 (5.0)	5 (12)	0	17	7 (41)
**Family history of glaucoma**	1 (1.1)	1 (1.1)	0	0	0	0	0	0	2	0

a = The total number of reasons does not match the total number of children, as a child may have more than one reason. The percentages are calculated per diagnosis.

b = Confirmed glaucoma include all patients, except patients who had the glaucoma diagnosis ruled out or who continued outpatient follow-up as suspected glaucoma.

GS= glaucoma suspect; PCG= Primary congenital glaucoma; JOAG= Juvenile open angle glaucoma; GNAOA= Glaucoma associated with non-acquired ocular anomalies; GAAC= Glaucoma associated with acquired conditions; GFCS= Glaucoma following cataract surgery; GNAS= Glaucoma associated with non-acquired systemic disease or syndrome.

In the group of patients with glaucoma following cataract surgery, congenital cataract surgery was performed without intraocular lens (IOL) implantation in 29 patients and with IOL implantation in nine. Table 3 shows the conditions related with glaucoma associated with non-acquired ocular anomalies, glaucoma associated with non-acquired systemic disease or syndrome, and glaucoma associated with acquired conditions.

**Table 3:** Distribution of conditions related with glaucoma associated with non-acquired ocular anomalies, glaucoma associated with acquired conditions and glaucoma associated with non-acquired systemic disease or syndrome

Final diagnosis	Associated conditions	n (%)
**GNAOA**	Peter’s anomaly	17 (63)
	Aniridia	2 (7.4)
	Anterior segment dysgenesis	2 (7.4)
	Axenfeld-Rieger syndrome	1(3.7)
	Iridocorneal cyst	1 (3.7)
	Eye abnormalities with indeterminate diagnoses	4 (15)
	**Total**	**27 (100)**
**GAAC**	Steroid	8 (40)
	Trauma	7 (35)
	Uveitis	4 (20)
	Retinal/choroidal detachment	1 (5.0)
	**Total**	**20 (100)**
**GNAS**	Sturge-Weber syndrome	4 (44)
	Noonan syndrome	1 (11)
	Neurofibromatosis	1 (11)
	Stickler syndrome	1 (11)
	Indeterminate systemic diseases or syndromes	2 (22)
	**Total**	**9 (100)**

GNAOA= Glaucoma associated with non-acquired ocular anomalies; GAAC= Glaucoma associated with acquired conditions; GNAS= Glaucoma associated with non-acquired systemic disease or syndrome.

Among 245 patients with glaucoma or suspected glaucoma,135 (55%) received the glaucoma diagnosis in the late period; 61 (25%), in the infantile period; and 49 (20%), in the neonatal period. The patients with primary congenital glaucoma had the shortest mean time to referral (9.2 months), of whom 50% were referred in the first 30 days of life and 98% were referred up to 2 years of age. Table 4 presents the distribution of the referral reasons per period in the patients with confirmed or suspected glaucoma.

**Table 4:** Distribution of referral reasons for neonatal, infantile and late periods in patients with confirmed or suspected glaucoma

Referral reasons	Neonatal	Infantile	Late
**Cup-to-disc ratio >0.5 or asymmetry** ≥**0.2, n (%)**	0	4 (7)	54 (93)
**IOP >21 mmHg, n (%)**	0	10 (14)	59 (86)
**Corneal opacity, n (%)**	37 (66)	16 (29)	3 (5.3)
**Myopic shift, n (%)**	0	7 (64)	4 (36)
**Buphtalmos, n (%)**	20 (43)	17 (37)	9 (20)
**Tearing, n (%)**	13 (46)	15 (54)	0
**Photophobia, n (%)**	8 (50)	8 (50)	0
**Family history of glaucoma, n (%)**	0	0	1 (100)
**Conditions at risk of developing glaucoma, n (%)**	0	7 (30)	16 (70)

Neonatal=until 30 days of life; Infantile= between 30 days and 2 years; Late= after two years.

The referral occurred after clinical surveillance for a known ocular disease in 38% of the children, after child health surveillance/screening in 31%, and owing to parental concern in 30%. Parents or caregivers were the first to identify the change and to seek for health care in 97% of the primary congenital glaucoma cases. Table 5 shows the persons who first detected the eye changes that led to the referral.

**Table 5:** Responsible for the first detection of eye abnormalities in each type of diagnosis

Final diagnosis	Parental concern^*^	Child health surveillance/ screening^**^	Clinical surveillance of children with known ocular disease^***^
**No glaucoma, n (%)**	10 (12)	49 (59)	24 (29)
**GS, n (%)**	5 (6)	44 (53)	34 (41)
**PCG, n (%)**	64 (97)	2 (3.0)	0
**JOAG, n (%)**	0	1 (50)	1 (50)
**GNAOA, n (%)**	10 (37)	0	17 (63)
**GAAC, n (%)**	1 (5.0)	4 (20)	15 (75)
**GFCS, n (%)**	4 (10)	1 (2.6)	33 (87)
**GNAS, n (%)**	5 (56)	1(11)	3 (33)
**TOTAL n (%)**	**99 (30)**	**102 (31)**	**127 (38)**

GS= glaucoma suspect; PCG= Primary congenital glaucoma; JOAG= Juvenile open angle glaucoma; GNAOA= Glaucoma associated with non-acquired ocular anomalies; GAAC= Glaucoma associated with acquired conditions; GFCS= Glaucoma following cataract surgery; GNAS= Glaucoma associated with non-acquired systemic disease or syndrome.

* Parents or caregivers were the first to detect eye abnormalities and seeked for health care.

** The child was referred by health carers (pediatricians, nurses, general ophthalmologists) in a routine evaluation.

*** General ophthalmologist referred children with known ocular disease in at-risk of glaucoma.

## DISCUSSION

In this study, we evaluated the referral reasons to a pediatric glaucoma outpatient clinic in a tertiary health-care service and the quality of screening by ophthalmologists and other health-care professionals. As visual prognosis depends on the time of diagnosis and subsequent treatment, it is crucial that health-care professionals are familiar with the relevant signs and need for referral. In more than half of the children referred to the glaucoma outpatient clinic, glaucoma was ruled out or suspect at the final diagnosis, with only 49% confirmed glaucoma cases. This fact indicates the importance and necessity of better training for glaucoma signs among the ophthalmologists and other health-care professionals who referred the patients.

Many conditions can simulate glaucoma, resulting in a high percentage of suspected or ruled out glaucoma as compared with confirmed glaucoma in the pediatric population. For example, while corneal opacity in infants may be a sign of glaucoma, other causes should also be considered, including corneal dystrophies, Peter’s anomaly, sclerocornea, dermoid, microphthalmia, birth trauma, metabolic disease (cystinosis and mucopolysaccharidosis). Obstetric trauma to the cornea can simulate Haab striae. Therefore, it is imperative for ophthalmologists to distinguish glaucomatous eyes from those with suspected glaucoma^(9,10)^.

As in other studies, most patients referred for evaluation at tertiary services in this study had suspected glaucoma, with the main reasons for referral being a CDR >0.5 or an asymmetry ≥0.2 and IOP >21 mmHg^(11-16)^. In the groups of children with a final diagnosis of suspected and no glaucoma, the most frequent reason for referral was CDR > 0.5 or asymmetry ≥0.2 (53% and 42%, respectively). While ninety-four children were referred on the basis of a CDR >0.5 or an asymmetry ≥0.2, only eight (8.5%) had a confirmed final diagnosis of glaucoma. Compared with the other referral reasons, CDR >0.5 or asymmetry ≥0.2 had a high rate of false-positive cases. Many conditions can simulate the appearance of the cupping of glaucoma, including macrodiscs/macrocups, periventricular leukomalacia, and congenital anomalies of the optic disc and axial myopia^(17-19)^. Although a CDR >0.5 or an asymmetry ≥0.2 was associated with many false-positive cases, the two cases with a final diagnosis of juvenile open-angle glaucoma were referred exclusively for these findings. Posteriorly, in the evaluation with childhood glaucoma consultants, both patients had an IOP >21 mmHg, but this parameter was not mentioned in the referral; it was not the reason for the referral. In addition, because children’s eye care does not routinely include tonometry, CDR >0.5 or asymmetry ≥0.2 may help in the detection of childhood glaucoma cases.

Corroborated by other studies, the children with final primary congenital glaucoma diagnosis were referred mainly for corneal opacity, buphthalmos, tearing, and photophobia^(1)^. In primary congenital glaucoma, corneal opacity is frequently the first sign recognized by parents or health professionals. As the referral is mostly dependent on external signs of the primary congenital glaucoma, the diagnosis of this group occurs most often as a result of parental concern. In this study, parents or caregivers were the first to identify the change and seek for health care in 97% of the cases^(4)^. As reported in another paper, the most common initial diagnostic sign in children diagnosed as having primary congenital glaucoma in the neonatal period was corneal opacity^(20)^.

Most patients (69%) were referred mainly through follow-up screening or clinical surveillance of children with ocular disease known to be associated with glaucoma, such as uveitis, aphakia, pseudophakia, Peter’s anomaly, aniridia, anterior segment dysgenesis, and Axenfeld-Rieger syndrome. Other conditions can also be associated with glaucoma, such as the Sturge-Weber syndrome, Noonan syndrome, neurofibromatosis, Stickler syndrome, and steroid use. These associations emphasize the need for close surveillance of at-risk children^(4)^. Therefore, pediatricians, health-care professionals, and general ophthalmologists must be aware of these associations and stimulate regular follow-up screening.

Although glaucoma was diagnosed on the basis of the CGRN classification^(8)^, no well standardized criteria have been established for children regarding IOPs >21 mmHg. Many studies have shown that the tonometry is affected by the central corneal thickness (CCT), with thicker corneas having artifactually high IOP readings, and children with ocular hypertension have higher CCTs^(21,22)^. Children who have undergone congenital cataract surgery can have higher CCTs and overestimated IOPs^(23)^. This fact may increase the number of referrals owing to the IOP overestimation. In our study, 50 (61%) of 82 children referred according to IOPs >21 mmHg had confirmed glaucoma diagnosis.

The other issues in our study relate to the fact that no standardized criteria have been established for referral to the pediatric glaucoma outpatient clinic in the tertiary health-care service from other less specialized services. The data obtained in this study call attention to the necessity of improving screening quality and awareness of referring screeners and providers.

All newborns are submitted for the red reflex test by a physician or other trained health-care professionals and evaluated within the first year of life by an ophthalmologist^(24)^. However, when optical changes are detected, such as corneal opacity, buphthalmos, tearing, photophobia, or abnormal red reflex test result, early evaluation by an ophthalmologist is mandatory. Early detection and treatment for childhood glaucoma improve the visual prognosis. Thus, ophthalmologists should measure the CDR and IOP in all children and evaluations (and when necessary, require an examination to measure the axial length). Children with conditions associated with glaucoma, such as phacomatoses (mainly Sturge-Weber syndrome), aphakia, pseudophakia, steroid use, ocular trauma, and Peter’s anomaly, must be evaluated at least every 6 months.

The three major causes of referral in our pediatric glaucoma outpatient clinic in a tertiary health-care service were CDR >0.5 or asymmetry >0.2, IOP >21 mmHg, and corneal opacity. However, the signs and referral reasons depending on age and diagnosis. In the neonatal period, the referral reasons were corneal opacity, buphthalmos, tearing, and photophobia, and parents or caregivers were the first to identify the changes in most cases. As these abnormalities are external and not so difficult to detect, it is important for pediatricians, health caregivers, and parents to be aware of these changes and seek early evaluation. Accordingly, greater dissemination of information on childhood glaucoma to the population and training for health professionals are important. After the neonatal period, besides these external changes, other signs were also reasons for referral, such as CDR >0.5 or asymmetry >0.2, IOP >21 mmHg, and myopic shift. The general ophthalmologist must evaluate these parameters, mainly in children at risk, such as those with aphakia, pseudophakia, steroid use, and Sturge-Weber syndrome. We suggest awareness of these findings to avoid and postpone diagnosis, identify their impacts on prognosis, and avoid spending important resources for the management of these diseases with inaccurate referrals.
